# Discovery and Characterization of Iron Sulfide and Polyphosphate Bodies Coexisting in* Archaeoglobus fulgidus* Cells

**DOI:** 10.1155/2016/4706532

**Published:** 2016-04-19

**Authors:** Daniel B. Toso, Muhammad Mohsin Javed, Elizabeth Czornyj, Robert P. Gunsalus, Z. Hong Zhou

**Affiliations:** ^1^Department of Microbiology, Immunology, and Molecular Genetics, UCLA, Los Angeles, CA 90095, USA; ^2^Electron Imaging Center for Nanomachines, California NanoSystems Institute, UCLA, Los Angeles, CA 90095, USA; ^3^The UCLA Biomedical Engineering Interdepartmental Program, UCLA, Los Angeles, CA 09905, USA; ^4^Institute of Industrial Biotechnology, GC University, Lahore 54000, Pakistan; ^5^The UCLA-DOE Institute of Genomics and Proteomics, UCLA, Los Angeles, CA 90095, USA

## Abstract

Inorganic storage granules have long been recognized in bacterial and eukaryotic cells but were only recently identified in archaeal cells. Here, we report the cellular organization and chemical compositions of storage granules in the Euryarchaeon*, Archaeoglobus fulgidus* strain VC16, a hyperthermophilic, anaerobic, and sulfate-reducing microorganism. Dense granules were apparent in* A. fulgidus* cells imaged by cryo electron microscopy (cryoEM) but not so by negative stain electron microscopy. Cryo electron tomography (cryoET) revealed that each cell contains one to several dense granules located near the cell membrane. Energy dispersive X-ray (EDX) spectroscopy and scanning transmission electron microscopy (STEM) show that, surprisingly, each cell contains not just one but often two types of granules with different elemental compositions. One type, named iron sulfide body (ISB), is composed mainly of the elements iron and sulfur plus copper; and the other one, called polyphosphate body (PPB), is composed of phosphorus and oxygen plus magnesium, calcium, and aluminum. PPBs are likely used for energy storage and/or metal sequestration/detoxification. ISBs could result from the reduction of sulfate to sulfide via anaerobic energy harvesting pathways and may be associated with energy and/or metal storage or detoxification. The exceptional ability of these archaeal cells to sequester different elements may have novel bioengineering applications.

## 1. Introduction

*Archaeoglobus fulgidus* strain VC16 is a hyperthermophilic, sulfur oxide-reducing, anaerobic archaeon. Belonging to the Archaeoglobales division of the Euryarchaeota, the species is commonly found in marine thermal vents, hot springs, and thermophilic oil field waters. The production of thiosulfate as well as hydrogen sulfide has been implicated in oil and gas souring and in oil pipeline corrosion [1, 2].* A. fulgidus* can produce biofilms in response to stress which may be important for metal detoxification, surface adherence, and nutrient acquisition [3]. Due to* A. fulgidus* being hyperthermophilic,* A. fulgidus* cells are used for metal sequestration in water treatment and serve as a source of high temperature stable enzymes.

*A. fulgidus* VC16 is able to grow chemoheterotrophically, thereby reducing sulfate. Initially isolated from marine hydrothermal vents in Italy [4, 5], it can utilize a variety of carbon compounds as electron donors for sulfate, as well sulfite and thiosulfate reduction to sulfide [6]. Some* A. fulgidus* strains are also capable of chemolithotrophic growth and use hydrogen as an electron donor with oxidized sulfur compounds as electron acceptors [7].* A. fulgidus* VC16 cells are morphologically spherical to irregularly coccoid in shape and some strains may be motile by appendages, possibly by flagella [5, 6].

In this study, we employ a combination of cryo electron microscopy (cryoEM), cryo electron tomography (cryoET), and electron dispersive X-ray (EDX) spectroscopy analyses to identify and characterize high-density inclusion bodies (also called granules) distributed within the cytoplasm of* A. fulgidus* VC16. We show that these structures are of two types which can each reach ~240 nm in diameter. One type is rich in compounds containing phosphorus and oxygen and the other in those containing iron and sulfur: both are typically positioned nearby or on the cell membrane and at opposite sides of the cell when the two types are present. Potential functions of these inclusion bodies include phosphate, iron, and sulfur deposits and energy storage in the form of polyphosphates and iron polysulfides, as well as metal sequestration in response to cell toxicity.

## 2. Materials and Methods

### 2.1. Cell Culture

*A. fulgidus* strain VC16 (DMS 4304) cells were cultured at 83°C in an anaerobic CO_2_/bicarbonate buffered mineral medium supplemented with vitamins and sodium lactate as previously described [8]. The medium contained per 1 L of ultrapure water 18 g NaCl, 3.4 g MgSO_4_-7H_2_O, 2.8 g MgCl_2_-6H_2_O, 0.5 g NH_4_Cl, 0.5 g KCl, 0.55 g KH_2_PO_4_, 0.14 g CaCl_2_-2H_2_O, 1 mL of a 1000x H^+^ trace mineral solution (50 mM HCl, 1 mM H_3_BO_3_, 7.5 mM FeCl_2_, 5 mM NiCl_2_, 0.5 mM MnCl_2_, 0.5 mM ZnCl_2_, 0.5 mM CoCl_2_, 0.5 mM CuCl_2_, 0.5 mM CuCl_2_, and 0.5 mM AlCl_2_), 1 mL of a 1000x OH trace mineral solution (10 mM NaOH, 0.1 mM Na_2_SeO_3_, 0.1 mM Na_2_WO_4_, and 0.1 mM Na_2_MoO_4_), and 1 mL of 1000x vitamin solution [9]. Sodium lactate was added to a final concentration of 20 mM. The medium was flushed with a N_2_/CO_2_ (80 : 20) gas mixture to remove oxygen and then dispensed into N_2_/CO_2_ flushed glass bottles. The bottles were then sealed with butyl rubber stoppers and crimp aluminum caps. The medium was autoclaved at 121°C. Prior to inoculation, the medium was supplemented with a sterile anaerobic stock solution of 2.5% Na_2_S-9H_2_O/2.5% Cysteine HCl (1% v/v) and 1 M NaHCO_3_ (2% v/v) to reduce the medium and adjust it to pH 7.0.

### 2.2. Preparation of* A. fulgidus* VC16 Cell Ghosts

Cells grown as described above (500 mL of culture) were divided into two equal portions and harvested by centrifugation at 5,000 ×g for 45 minutes at room temperature. Pellets were resuspended in 1 mL of “Wash Buffer” (18 g L^−1^ NaCl, 3.4 g L^−1^ MgSO_4_-7H_2_O, 2.8 g L^−1^ MgCl_2_-6H_2_O, 0.147 g L^−1^ CaCl_2_-2H_2_O, 20 mM KH_2_PO_4_, adjusted to pH 7 with NaOH) and transferred to a 2 mL microfuge tube. The cells were centrifuged at 12,300 ×g for 1 minute and the pellet was washed three additional times in 1 mL of Wash Buffer. The pellet was resuspended by gentle pipetting. After the final wash, the cell pellets were suspended in 1 mL of “Wash Buffer” containing 1 *μ*L of DNAse (10 *μ*g/mL) and 1 *μ*L of RNAse (10 *μ*g/mL). This cell suspension was then immediately frozen in a dry ice/ethanol bath. Immediately after freezing it was then allowed to completely thaw in a water bath at room temperature. The freeze/thaw step was repeated 5 additional times. The cell suspension was then centrifuged at 12,300 ×g for one minute and the supernatant was transferred to a 2 mL microfuge tube.

The “cell ghost” pellet was washed three times in a “Low Salt Buffer” (100 mM Tris pH7 adjusted with HCl, 20 mM MgSO_4_, 4 mM CaCl_2_) by suspending the pellet in 1 mL by pipetting up and down. The cells were then centrifuged at 12,300 ×g for 1 minute. Once the pellet was washed three times, it was then suspended in 1 mL of “Low Salt Buffer” and stored at −70°C or used directly for cryoEM.

### 2.3. Electron Microscopy

For negative stain transmission electron microscopy (TEM), copper grids with carbon-coated Formvar film were glow-discharged and followed by placement of a small droplet (~4 *μ*L) of* A. fulgidus* cell suspension onto the carbon side of each grid. After one minute at room temperature, the excess of the sample droplet was blotted from the side of the grid and a droplet of 1% uranyl acetate solution was immediately placed on the grid. After the stain sat for one minute, it was blotted off and the staining/blotting step was repeated four times to remove any excess sample from the grid. After air drying, the grid was observed in an FEI Tecnai F20 transmission electron microscope operated at an accelerating voltage of 200 kV to assess the concentration of cells on the grid. To adjust concentration, cells were centrifuged at low speed (12,300 ×g) in a table-top microfuge and resuspended in culture medium.

To prepare frozen hydrated cells for cryoEM and cryoET, 100 *μ*L of cell solution with optimized concentration was mixed with 4 *μ*L of 10 nm protein-A gold solution and mixed thoroughly. A droplet (4 *μ*L) of this mixed solution was placed onto a glow-discharged, 200 mesh Quantifoil holey carbon grid with spacing of 3.5 *μ*m hole/1 *μ*m edge. The sample was allowed to settle for 30 seconds, blotted by filter paper, and immediately plunged into liquid nitrogen cooled liquid ethane to produce frozen hydrated samples. Samples were loaded into a Gatan 626 cryosample holder and low-dose cryoEM images were recorded in the above-mentioned Tecnai F20 electron microscope on a 16-megapixel TVIPS CCD camera.

### 2.4. Cryo Electron Tomography

For collecting the cryoET tilt series, we used an FEI Titan Krios instrument operated at an accelerating voltage of 300 kV and equipped with a Gatan image filter (GIF) 2002 camera. During tilt series acquisition, we chose cells whose long axes were roughly parallel to the tilt axis of the microscope. The cells were imaged at ~30,000x magnification. An underfocus value of 6 *μ*m was targeted for all images in the tilt series. All tomography tilt series were recorded using the FEI Batch Tomography software with a tilt range from −70° to +70° and 2° increment. The total electron dosage on the sample is 200 e^−^/Å^2^ for each tilt series.

### 2.5. CryoET Data Analysis and 3D Visualization

The tomography tilt series were processed with a suite of programs to generate 3D reconstructions. Alignment of the tilt series was performed using the eTomo tomography processing software from the* Imod* package [10]. The steps included X-ray removal, rough alignment by cross-correlation, fine alignment by fiducial gold tracking, and tilt axis adjustment. The aligned tilt series were then used to make 3D reconstructions using weighted back-projection reconstruction in* eTomo* or GPU-based SIRT (Simultaneous Iterative Reconstruction Technique) reconstruction implemented in Inspect3D. The 3D reconstructions were saved as stacks of* X-Y* plane images that are single pixel slices along the *Z*-plane. Slices from the reconstructions were displayed using slicer within 3dmod from the* Imod* package. Amira (Visage Imaging GmbH, http://www.amira.com/) was used to create volume renderings of the 3D density maps of the cells.

### 2.6. STEM and EDX Analysis

STEM imaging and EDX analysis were performed using an FEI Titan 80–300 kV scanning transmission electron microscope.* A. fulgidus* cells were placed on carbon-coated copper grids, air-dried, and imaged inside the Titan instrument. The STEM images were collected on a HAADF detector.

FEI TEM Imaging and Analysis (TIA) software package was used to acquire the line scan and area scan EDX spectra of different areas of the sample at 300 kV. The spectral data for the indicated elements were stored as counts over distance or area. The TIA software package was used to output the individual point spectra as well as to generate the line scan and area plots.

## 3. Results

### 3.1. Imaging of* A. fulgidus* VC16 Whole Cells and Cell Ghosts

**Figure 1:**
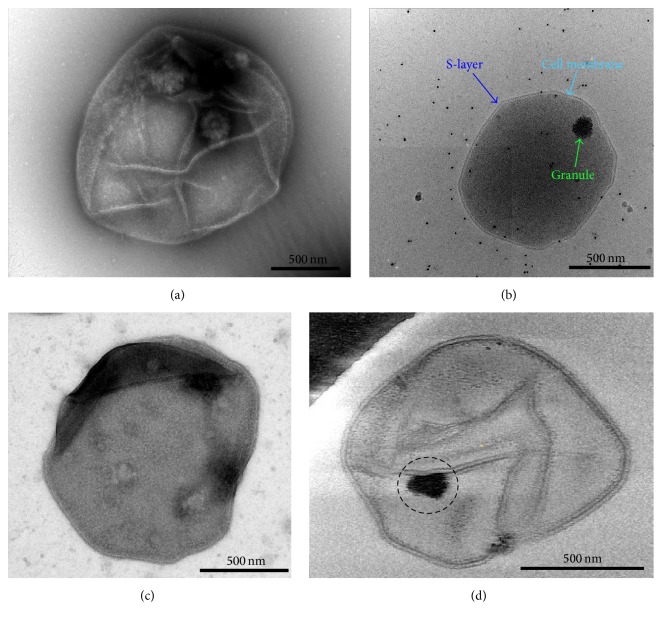
Electron microscopy of* A. fulgidus* cells and visualization of intracellular granules. (a, b) Transmission electron microscopy images of an* A. fulgidus* cell stained by 1% uranyl acetate (UA) (a) or embedded in vitreous ice (b). (c, d) Representative TEM images of cell ghosts of* A. fulgidus* stained with UA (c) or embedded in vitreous ice (d). The remains from a possible dense granule are circled (d).

Initially, we imaged whole* A. fulgidus* cells negatively stained with 1% uranyl acetate (UA) by conventional TEM (Figure 1(a)). However, the large size and thickness of whole cells prevented stain penetration and thus complicated the visualization of the interior of the cell. Consequently, negative stain TEM images did not routinely show evidence for the existence of dense granules within the cells. By contrast, cells embedded in vitreous ice directly imaged by cryoEM without staining clearly reveal the presence of one or more electron dense granules in each cell (Figure 1(b)), despite intrinsic low contrast of the cryoEM images owing to the use of low-dose exposure necessary to minimize radiation damage to the cell.

To reduce thickness of the sample and improve staining, cell ghost preparations were made from whole* A. fulgidus* cells as described in Section 2 and imaged after being negatively stained with a 1% UA solution (Figure 1(c)). The ordered S-layer coat surrounding the cell membrane is apparent [11], whereas the granules usually present within either were absent or did not appear as uniformly dark electron dense bodies. This result may be due to loss of structural integrity during ghost preparation process, whereby most of the cellular cytoplasm is extracted leaving behind the cell membrane with attached S-layer proteins. While this preparation leaves behind cell ghosts that are easily stained and flatten on the carbon support film of the grid for proper examination by TEM, the loss of cell contents does not allow consistent visualization of the dense granules.

To overcome these difficulties with negative stain TEM, we imaged the same cell ghost preparation by cryoEM. The* A. fulgidus* cell ghosts imaged this way also had very poor contrast due to the thinness of the sample, so electron tomograms were reconstructed after collecting tilt series by cryoET (Figure 1(d)). What appear to be remnants of the granules, possibly attached to the cellular membrane, were occasionally visualized within the cell ghosts (circled in Figure 1(d)). There also appear to be cell envelope-associated structures or scars, which may have been granule assembly sites (Sup. Figure S1, arrows, in Supplementary Material available online at http://dx.doi.org/10.1155/2016/4706532). Imaging this preparation, however, did not allow routine visualization of intact dense granules within the cell, as most of the cellular contents were lost during sample preparation.

### 3.2. 3D Visualization of Inclusion Bodies within* A. fulgidus* VC16 Cells by CryoET

**Figure 2:**
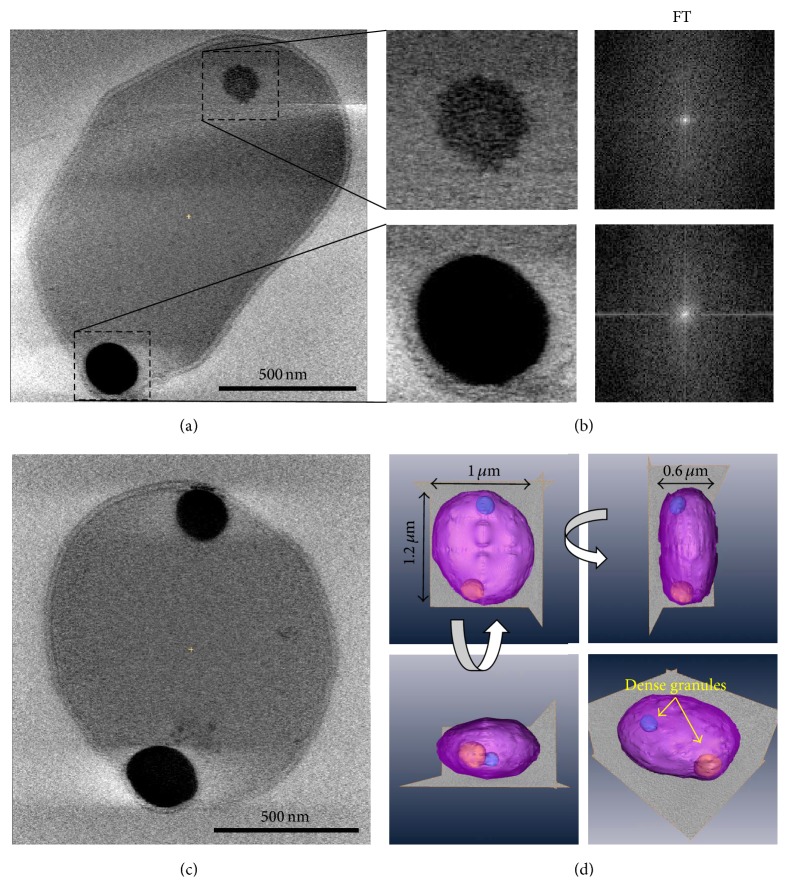
CryoET of* A. fulgidus* cells reveals dense cytoplasmic bodies. (a) Central slice from a 3D tomogram reconstructed from a cryoET tilt series of a whole cell frozen in vitreous ice. (b) The two granules present in (a) are enlarged to show their amorphous features. The Fourier transform of each granule is shown on the right. (c) Central slice from the 3D tomogram of another cell. (d) 3D rendering of the cell in (c) shown from different angles. The cell is shown in pink, the 2 granules are shown in blue and yellow, and the dimensions of the cell are indicated.

Next, we sought out to use cryoET to reconstruct structures of whole cells in three dimensions (Figure 2). Embedding whole cells in vitreous ice preserves their structures in a natural state and eliminates the artifacts associated with staining (Figure 2(a)). These cryoET reconstructions of* A. fulgidus* cells reveal typical S-layer envelope surrounding the cellular monolayer membrane composed of C40 isoprenoid diether lipids [11, 12]. The reconstructions also show that the* A. fulgidus* cells are coccoid to irregular in shape and approximately 1 *μ*m in diameter. The clearly visible cell membrane layer (~37 Å thick) is surrounded by a uniform protein S-layer (~110 Å thick). These two structures are separated by a periplasmic-like space (~130 Å) (Figure 2(a)). The most apparent feature within the cytoplasm is the presence of one or two electron dense inclusion bodies. The presence of these high-density inclusion bodies has not been previously described for* A. fulgidus*. The granules vary from roughly spherical to ellipsoidal with smooth rather than angular surfaces (Figures 2(a)–2(c)).

Based on density, these granules can be classified as nascent and developed granules. Nascent granules are frequently observed within cells undergoing exponential growth phase (Sup. Figure S2). They appear to be composed of very small particles which have clustered into a closely packed, roughly spherical shape (Figure 2(a), upper granule). Developed granules are more rounded and do not exhibit the particulate appearance (Figure 2(a), lower granule, and Figure 2(c)) and are found in stationary phase cells. No repeating/crystalline or ordered arrangements either were observed within granules visually in the packing or are apparent from the Fourier transform (FT) of the images of the inclusion bodies (Figure 2(b)). The lack of crystalline or ordered arrangement indicates that the bodies have an amorphous arrangement. Inspection of freeze-thaw prepared cell ghosts reveals apparent disintegration of granules as evidenced by many small particulate fragments (Figure 1(d)).

**Figure 3:**
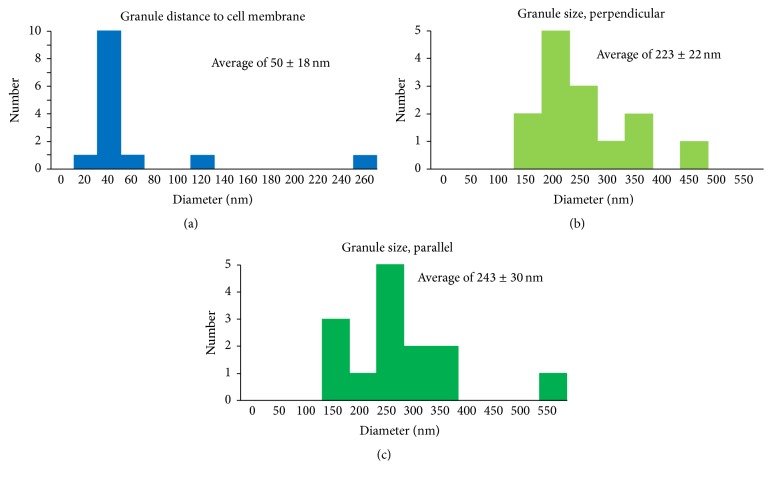
Size and location distribution of granules in* A. fulgidus* cells. (a) Distance of granules from the cell membrane. (b) Diameter of granules perpendicular to the cell membrane. (c) Diameter of granules parallel to the cell membrane.

Cells in stationary phase exhibited a characteristic arrangement in which one or two dense, developed granules are present on the periphery of the cell (Figure 2(c)). When these cells are reconstructed in three dimensions by cryoET, it is further observed that the granules are positioned very close to the cell membrane, often appearing to touch the surface of the cell and causing local membrane deformations (Figure 2(d)). By examining the 3D tomograms of cells containing the dense bodies we obtained the following statistics of size and localization distribution. The granules are positioned close to the edges of the cell, with an average center distance of around 50 (±18) nm from the cell membrane (Figure 3(a)). Their shape appears to be ellipsoidal, with a size distribution slightly longer along the axis of the cell membrane with an average size of 243 (±30) nm parallel to the membrane and 223 (±22) nm perpendicular to the membrane (Figures 3(b) and 3(c)). This statistical analysis confirms our structural observation that the granules are specifically localized near the membrane and often locally deform the cell envelope surface (by ~20–30 nm). This observation suggests that the granules may be associated with one or more membrane activities within the cell (discussed below).

### 3.3. Elemental Composition of Granules

**Figure 4:**
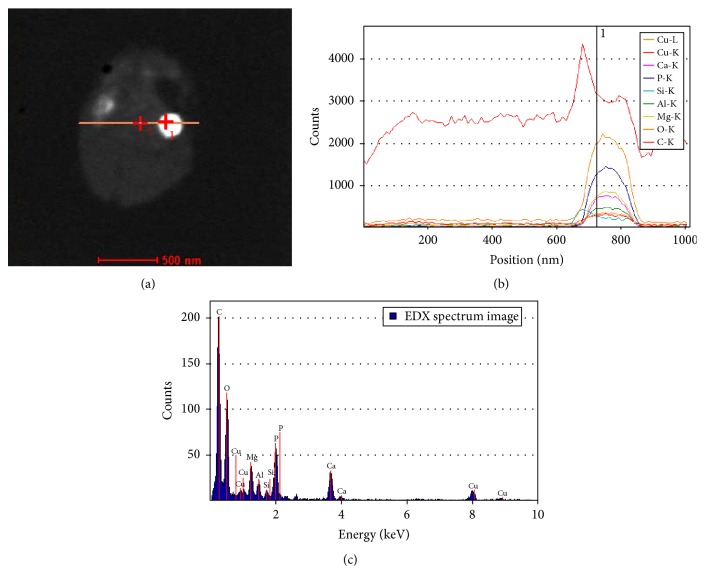
Elemental composition through a granule cross section by EDX analysis. (a) STEM image of the analyzed cell; the path of the probe is shown as an orange line. (b) The EDX line spectra across the cell shown in (a). Each element is plotted in a different color. (c) The individual EDX point spectrum from the location specified in (a) by a cross with the number 1 and in (b) by a vertical line.

Using an FEI Titan STEM instrument, we collected EDX spectra to analyze the elemental composition of the inclusion bodies within representative* A. fulgidus* VC16 cells. Initially, element line spectra across the* A. fulgidus* bodies were obtained by scanning the electron probe along a line across the sample, and the spectra revealed high abundance of phosphorus and oxygen within the granule region (Figures 4(a) and 4(b)). Individual point spectra in the region of the granule show that, in addition to phosphorus and oxygen, calcium, magnesium, copper, and aluminum are also concentrated in the dense bodies (Figure 4(c)). The high concentration of phosphorus and oxygen along with the cationic elements is consistent with our previous results demonstrating the presence of polyphosphate bodies (PPBs) in* Methanospirillum hungatei* JF1 [13]. The demonstration of these very concentrated PPBs in a third Euryarchaeota species was encouraging, but upon further EDX analysis we found some of the granules had very different spectra.

**Figure 5:**
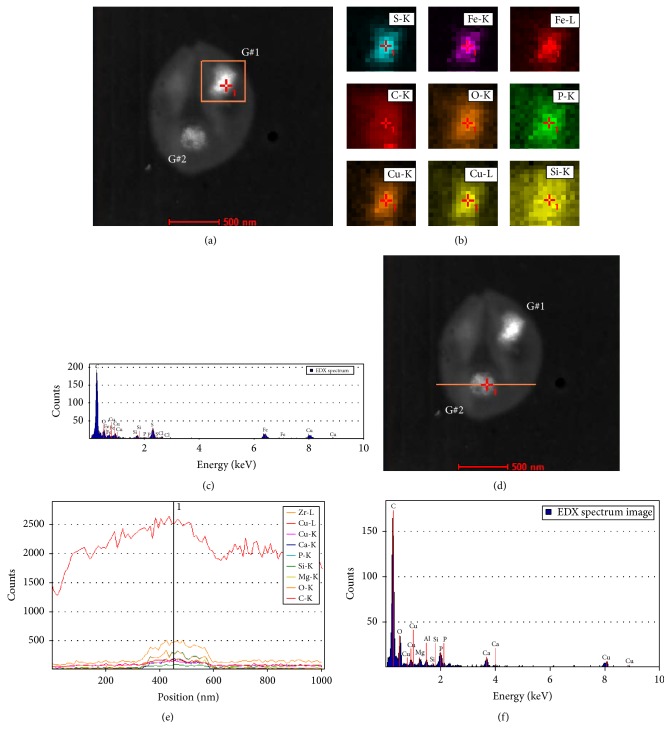
Separate EDX analysis of two granules in a single intact* A. fulgidus* cell. (a) STEM image of the analyzed cell; the first area scanned by the probe is shown as an orange box. The two granules are labeled as G#1 and G#2. (b) The EDX area scans for each element found in the region specified in (a), which contains G#1. Each element is plotted in a different color. (c) The individual EDX point spectrum from the location specified in (a) and (b) by a cross with the number 1. (d) STEM image of the analyzed cell; the path of the probe for the second scan is shown as an orange line. (e) The EDX line spectra across the cell shown in (d), which contains G#2. Each element is plotted in a different color. (f) The individual EDX point spectrum from the location specified in (d) by a cross with the number 1 and in (e) by a vertical line.

Area scan spectra were obtained through dot mapping by scanning specific areas of additional cells which included one or more inclusion bodies: some of these area scans showed high concentrations of iron and sulfur, not phosphorus and oxygen, within the bodies (Figures 5(a) and 5(b)). Examining individual point spectra in the region of these new granules showed that sulfur and iron were highly concentrated along with copper. In contrast, phosphorus and oxygen plus associated magnesium, calcium, and aluminum were not concentrated relative to surrounding cellular cytoplasm (Figure 5(c)). This new type of granule, named iron sulfur bodies (ISBs), in which iron and sulfur are concentrated, is suspected to be composed of ferrous sulfide (Fe-S), ferrous thiosulfate (FeS_2_O_3_^2−^), or possibly Fe-polysulfide (Fe-[S_*n*_]). These would occur in the reduction of sulfate (SO_4_^2−^) to hydrogen sulfide (H_2_S) during the crucial anaerobic respiration process needed for cell energy production and survival. Conceivably, ferrous thiosulfate or Fe-polysulfide would be formed as intermediates of the sulfate reduction pathway to serve as energy conserving storage materials. The ISB would subsequently be reduced to sulfide (HS^−^) for energy harvesting in downstream pathway reactions. The biological formation of thiosulfate from sulfite has been reported in several sulfate-reducing bacteria including* Desulfovibrio vulgaris, Desulfovibrio desulfuricans,* and* Thermodesulfobacterium commune,* whereby the enzyme systems responsible are not well described [14–16]. These dense granules containing high concentrations of iron and sulfur may serve alternatively in metal storage and/or metal detoxification roles by analogy to PPBs in bacteria [17–19].

**Figure 6:**
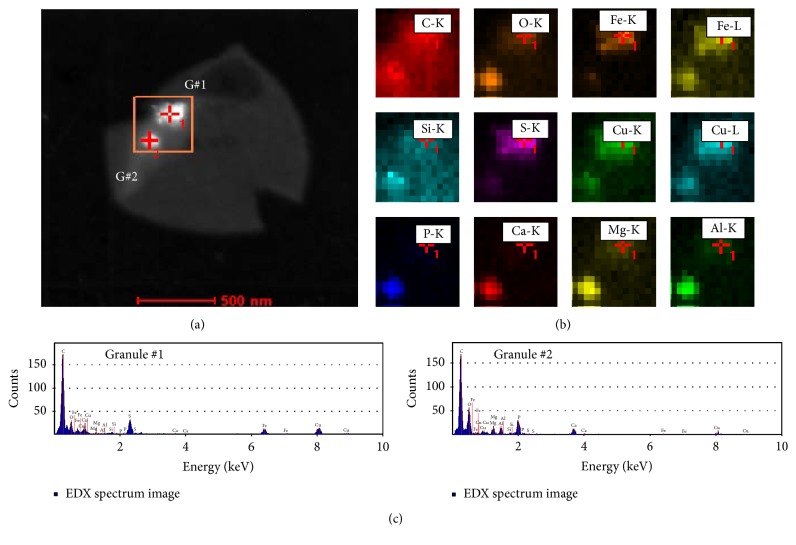
Combined EDX analysis of two granules in a single* A. fulgidus* cell. (a) STEM image of the analyzed cell; the area scanned by the probe is shown as an orange box. The two granules are labeled as G#1 and G#2. (b) The EDX area scans for each element found in the region specified in (a), which contains both G#1 and G#2. Each element is plotted in a different color. (c) The individual EDX point spectra from the locations specified in (a) by crosses with the numbers 1 and 2. The first spectrum is from G#1 and the second one is from G#2.

In many* A. fulgidus* cells two granules were discovered to be present (Figures 2, 4, 5, and 6). When granules were found which contained high concentrations of iron and sulfur and were identified as ISBs (Figures 5(a)–5(c)) the second granule in these cells was subsequently analyzed and established to be composed of elevated concentrations of phosphorus and oxygen along with calcium, magnesium, copper, and aluminum, therefore identified as PPBs (Figures 5(d) and 5(e)). Line scan spectra across the cell and examination of the individual point spectra in the granule region showed the characteristic PPB elemental composition seen before (Figure 5(f)). The individual colocalization of the two types of granules within the same cell was repeatedly observed.

In order to confirm the colocalization of granule types, area scan spectra were performed which contained both granules within the same cell. The area scans again revealed that the granules had very different compositions, one contained high concentrations of sulfur and iron, while the other had high concentrations of phosphorus and oxygen (Figures 6(a) and 6(b)). Contrasting the individual point spectra from within each granule in the cell confirmed the very different compositions and showed the characteristic spectra shown previously for ISBs and PPBs in this cell type (Figure 6(c)).

### 3.4. Effect of Cell Nutrition on Dense Body Formation

We further showed that the ability of* A. fulgidus* VC16 cells to form the two body types depends, in part, on the composition of the cell culture medium. When phosphate was limited, no dense bodies were formed. The main effect of limiting specific nutrients in the culture medium is manifested in the cell morphology. Reduced phosphate and lactate caused cells to grow to be less dense and smaller in size when observed in negative stain (Sup. Figure S3). The absence of phosphate in culture media caused cells to appear further shrunken (Sup. Figure S3 panel E). After treating for three generations under the altered conditions, having no phosphate in solution did not allow cells to proliferate (Sup. Figure S4). Though possible artifacts associated with negative stain TEM complicate these observations, cryoEM images of cells with reduced phosphate show a clear reduction in size compared to normal concentrations.

## 4. Discussion

Intracellular granules have been characterized in bacteria, eukaryotes, and archaea utilizing both energy-filtered TEM and energy dispersive X-ray spectroscopy in order to analyze the elemental composition of the inclusion bodies [13, 20–22]. Our current study demonstrates the coexistence of two types of granules, PPB and ISB, in the same* A. fulgidus* VC16 cell located near cell membranes. Notably, the PPBs lack the iron and sulfur elements abundant in the ISBs, while the ISBs lack the phosphate and oxygen abundant in the PPBs. Likewise, the less abundant elements present in the PPBs (magnesium, calcium, and aluminum) are low to absent in the Fe-S bodies and, conversely, the PPBs lack iron. The ratios of the predominant elements seen in PPBs and ISBs are diagnostic: the former exhibits a characteristic oxygen, phosphorus, iron, sulfur ratio of 2 : 1 : 0 : 0, while the latter has an elemental ratio of 0 : 0 : 1 : 2. This elemental analysis also provides a potential approach to high throughput STEM assisted cell screening to characterize inorganic granules in* A. fulgidus* and in other archaeal and bacterial strains. Further analyses of the variations in granule density and at different stages of development are also now possible.

Both types of granules (PPBs and ISBs) within* A. fulgidus* strain VC16 are positioned in characteristic membrane-adjacent locations (Figures 2–4). These uniform locations suggest a means to position each granule type within the cell along with the genetic ability to spatially and temporally program granule formation. The precise positioning of granules is also found in a magnetotatic bacterium which produces a magnetosome structure composed of arranged magnetic granules, though there is no evidence for the formation of ISBs or PPBs via invagination of the inner membrane or specific associated proteins, as found with the magnetosome [23, 24]. The effect of cell nutrition on* A. fulgidus* VC16 granule formation was also examined where limiting the carbon or phosphate supply resulted in formation of no PPBs or ISBs per cell. This observation is consistent with the ability of the cell to monitor environmental conditions and control elemental sequestration accordingly.

Potential roles of the PPB granules in* A. fulgidus* were mentioned above based on prior PPB studies in the bacteria and eukaryotes over the past thirty years [17–19, 25]. Besides roles in phosphate storage and cell energy capture, other PPB functions include roles in chromosome replication, cell division, metal chelation, and metal detoxification.

The role(s) of the newly described ISB granules in* A. fulgidus* are unknown. Besides potential roles in iron or sulfur-based energy storage, ISB granules may also have roles in metal sequestration and/or detoxification by analogy to the PPBs.* A. fulgidus* species thrive in highly reducing and metal rich environments. Fluids flowing from hydrothermal vents, for example, from black and white smoker vents, are reported to contain dissolved calcium, copper, zinc, iron, manganese, and strontium in the low to high micromolar range [26]. Nearby sea waters rich in dissolved magnesium, phosphate, and sulfate recirculate within these vent fluids which would supply primary sources of phosphorus, sulfur, and other metal cations. These black and white smokers also have other associated metal precipitates and soluble by-products that may be toxic to nearby microbes. The ecology of these habitats is relatively unstudied.

As shown in Figures 5 and 6, the PPB and ISB granules are positioned in characteristic cell locations nearby or on the cytoplasmic membrane surface. This location suggests that the cell possess a genetic means to initiate development of each granule type in a spatial and temporal context. This membrane proximity would presumably facilitate accumulation of nutrients from the environmental surroundings for chemical storage and utilization as cell reserves. We expect that enzyme machinery to facilitate PPB and ISB formation resides at or nearby the cell membrane. PPBs near cell membrane could coordinate accumulation of phosphate from the cell exterior via high affinity uptake systems (e.g., AF0791, AF1356–1360, and AF1798) with colocalized polyphosphate polymerizing enzymes nearby for granule assembly. The presence of structures or scars visualized along the inner surface of the cytoplasmic membrane supports the notion of associated enzyme machineries (Sup. Figure S1). For example, polysulfur and/or iron depositing enzymes would be associated with the ISB granules. It was previously shown that* A. fulgidus* cells metabolize sulfate and sulfite as well as thiosulfate [6], and the pathway intermediates leading to sulfide production could provide potential substrates for granule formation. The genome contains two uptake systems for iron 2 (AF0246 and AF2394) and for iron 3 (AF 04302 and AF1401-1402), a highly unique iron storage ferritin (AF0834), a P-type copper transporter (AF1052), and a copper chaperone (AF0346), plus one sulfate ABC-type system (AF00923). Tests of their annotated roles and means of granule formation await the development of genetic tools.

From the* A. fulgidus* VC16 cryoEM measurements of cell envelope and granule dimensions we can accurately document the cell compartment volumes and surface areas. A spherical cell of one micron in diameter would have an overall cell volume of 0.524 *μ*m^3^ (volume, *V* = (4/3)*πr* ^3^). Using the following measures of the cell membrane cross section (~37 angstroms thick), the S-layer lattice (~110 angstroms thick), and the periplasmic-like space (~130 angstroms thick) which is sandwiched between and separates the two structures (Figure 2(a)), the* A. fulgidus* volume is partitioned into 84.3% cytoplasm (0.441 *μ*m^3^), 2.1% cell membrane (0.011 *μ*m^3^), 7.2% cell periplasm (0.038 *μ*m^3^), and 6.4% cell S-layer (0.034 *μ*m^3^). The intracellular PPB and ISB granules observed in* A. fulgidus* cells can individually compose up to 1.4% of the cytoplasmic space (~230 nm diameter). Assuming the PPB granule density reported by Toso et al. [13], a single* A. fulgidus* PPB would store several-hundred-fold more energy in the form of phosphoanhydride bond energy than contained in the cellular ATP pool.

Compared to the cross section of a typical* E. coli* cell envelope (~29 nm thick including the CM, periplasmic space, and outer membrane (OM)) the analogous* A. fulgidus* envelope dimensions are remarkably similar (~28 nm thick) [27]. Here, the S-layer lattice (~11 nm in cross section) replaces the bacterial peptidoglycan and OM layers (~6.9 nm for the* E. coli* OM) and may contribute to cell rigidity and shape [28]. The corresponding cross section dimensions of the archaeal and* E. coli* periplasmic spaces also differ by about 25% (~13 nm in* A. fulgidus* versus ~16 nm in* E. coli*). They provide analogous roles while having remarkably different cell architectures and molecular compositions.

Few examples of inorganic storage granules are reported in archaea in contrast to PPB granules in bacteria and eukaryotes [17–19]. We recently described the presence of PPBs in the methanogen* Methanospirillum hungatei* JF1 [13] which contained spherical granules of approximately 150 nm in diameter. They were positioned along the central axis of the cell and away from the cell membrane relative to* A. fulgidus* granules. The* M. hungatei* PPBs bodies also differed from* A. fulgidus* PPBs reported in this study, whereby the* M. hungatei* bodies contain iron plus calcium rather than magnesium, aluminum, copper, and calcium (Figure 6(c)). These data establish subclasses of PPBs in archaea which differ in the types of cations accumulated. Additionally, since* A. fulgidus* VC16 cells can possess both PPBs and ISBs, it is evident that this archaean can somehow discriminate between available cations and selectively incorporate them into their two granule types (e.g., the PPBs lack iron, while the ISBs lack Ca, Al, and Mg).

Reports of PPB-like structures in other archaeal genera include several strains of* Sulfolobus* and* Methanosarcina* [20, 22]. This study is the first to report ISBs in archaea, although formation of polysulfides and polythionates has been described in the phototrophic purple sulfur bacteria (e.g., Chromatiaceae and* Ectothiorhodospira* species [29, 30]), where reduced sulfur compounds are oxidized as electron donors during anoxygenic light energy harvesting. In the chemoautotrophic species* Thiobacillus ferrooxidans,* “sulfur globules” were reported to contain an inner core of S_7–12_ polysulfur plus an outer layer of S_19+_ polythionates [31]. The molecular composition of the* A. fulgidus VC16* ISBs is currently unknown. Inspection of negatively stained cell sections of* A. fulgidus* strain 7324, which is related to* A. fulgidus VC16*, reveals the presence of electron dense bodies nearby the cell membranes (Figure  1; [1]). Although not described by the authors, these dark bodies likely contain polyphosphate (PPBs) and/or Fe-S (ISBs) reported in this study. Future investigations are needed to understand the nutritional, biochemical, and genetic basis for PPB and ISB granule formation in archaea as well as their physiological roles in cell metabolism/detoxification.

## 5. Conclusion

The occurrence, location, size, and compositions of two types of intracellular bodies in the thermophilic archaean* Archaeoglobus fulgidus* VC16 are demonstrated for the first time. Each is composed of distinct primary and secondary metals and is likely involved in nutrient and/or energy storage. The presence of polyphosphate bodies in the archaea along with bacteria and Eukarya suggests an ancient origin of these structures. Future studies are needed to explore the biogenesis and physiological uses of these inclusion bodies.

## Supplementary Material

Supplementary Figure S1: Negative stained *A. fulgidus* cell ghosts reveal “scars” along cell membrane. Arrows indicate the location of “scars” found in *A. fulgidus* cell ghosts.
Supplementary Figure S2: *A. fulgidus* cells in exponential growth phase showing newly developed granules. A,B) Slices from 3D tomograms reconstructed from cryoET tilt series of *A. fulgidus* whole cells in growth phase. The insets show the granules present within each cell at higher magnification.
Supplementary Figure S3: *A. fulgidus* development under varying nutritional conditions. A-E) TEM images of (A) a cell grown in normal conditions, (B) a cell grown with excess phosphate, (C) a cell grown with reduced phosphate, (D) a cell grown with reduced lactate, and (E) a cell grown with no phosphate. All samples were negatively stained with 1% UA.
Supplementary Figure S4: CryoEM of *A. fulgidus* cells after three generations of reduced or elevated phosphate conditions. A) Low magnification image of a TEM grid, stained with 1% UA, of a sample grown with no phosphate. Almost no cells are present. B-E) CryoEM images of cells grown in (B) 1 mM phosphate, (C) 2 mM phosphate, (D) 4 mM phosphate (normal conditions), and (E) 9 mM phosphate.
