# Supplementary material for: Discovery and Characterization of Iron Sulfide and Polyphosphate Bodies Coexisting in Archaeoglobus fulgidus Cells
Source: Archaea. 2016 Apr 19;2016:4706532. doi: 10.1155/2016/4706532 (PMC4853940; doi:10.1155/2016/4706532)

Supplementary Figure 1

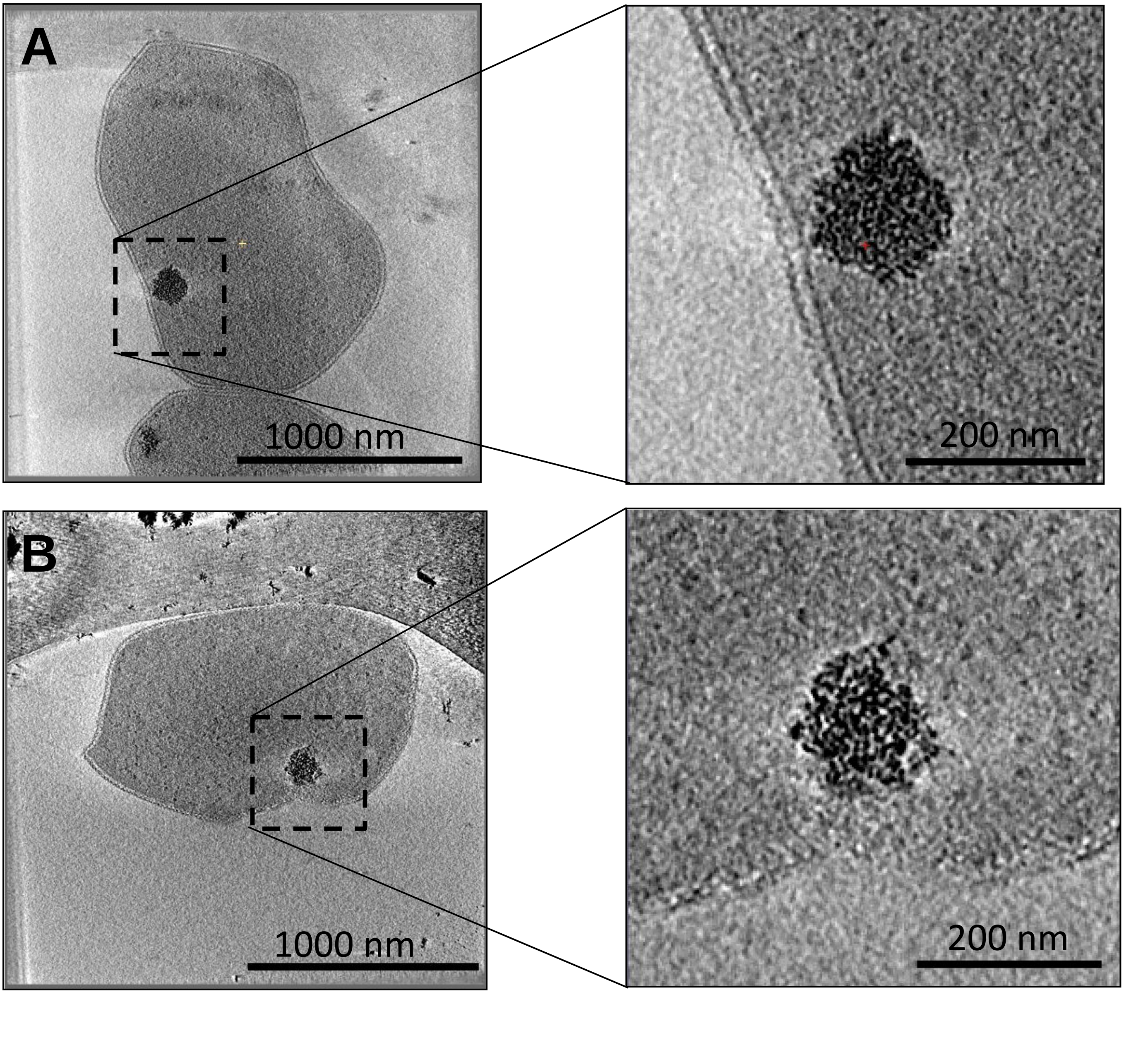

Supplementary Figure 2

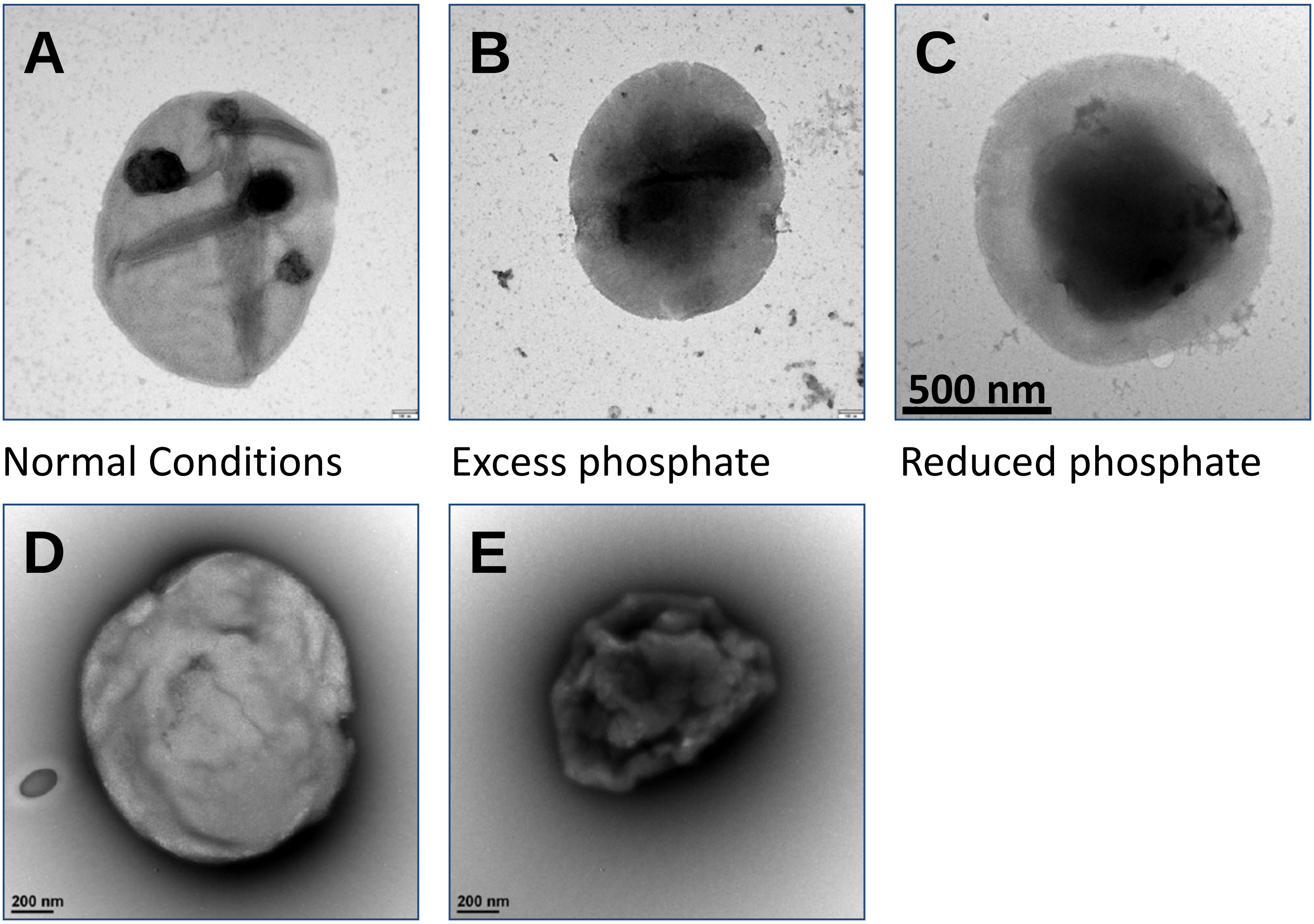

A: Normal Conditions

B: Excess phosphate

C: Reduced phosphate

D: Reduced lactate

E: No phosphate

Supplementary Figure 3

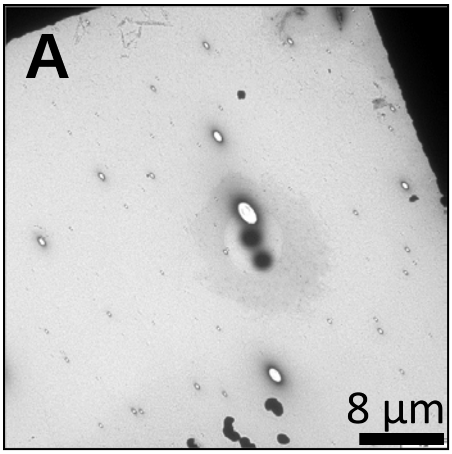

0 mM PO_4

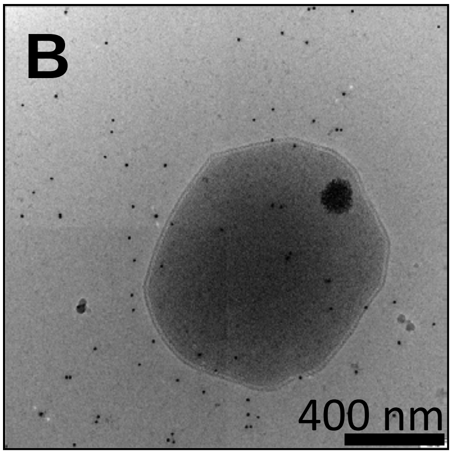

1 mM PO_4

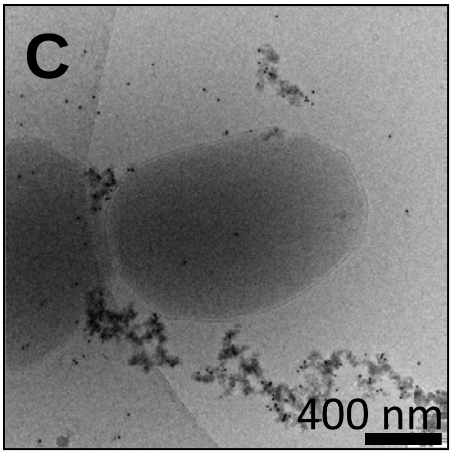

2 mM PO_4

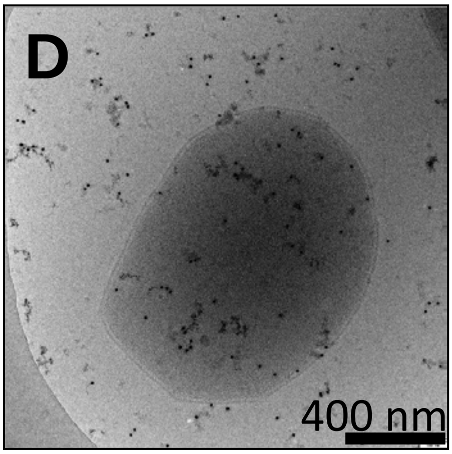

4 mM PO_4

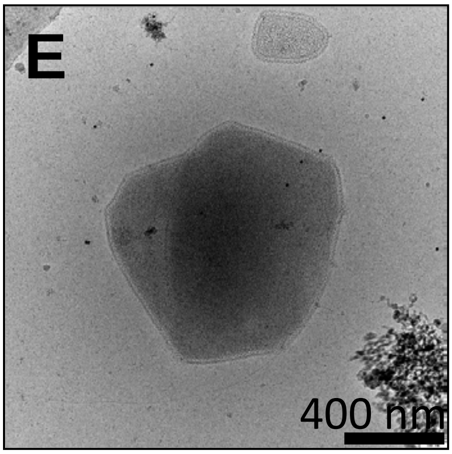

9 mM PO_4

Supplementary Figure 4

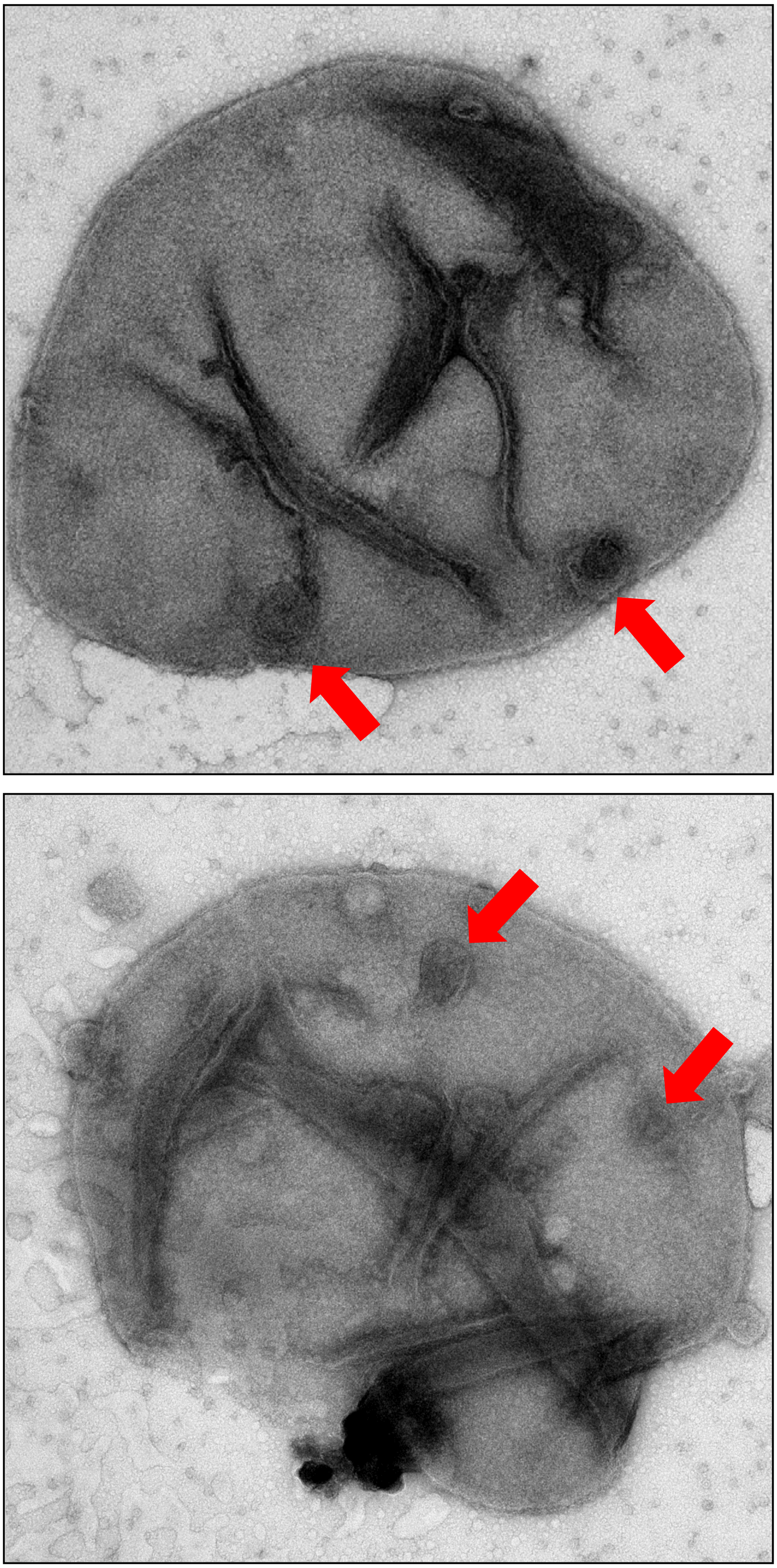

Supplement: Supplementary file 1 — Supplementary Figure S1: Negative stained A. fulgidus cell ghosts reveal “scars” along cell membrane. Arrows indicate the location of “scars” found in A. fulgidus cell ghosts. Supplementary Figure S2: A. fulgidus cells in exponential growth phase showing newly developed granules. A,B) Slices from 3D tomograms reconstructed from cryoET tilt series of A. fulgidus whole cells in growth phase. The insets show the granules present within each cell at higher magnification. Supplementary Figure S3: A. fulgidus development under varying nutritional conditions. A-E) TEM images of (A) a cell grown in normal conditions, (B) a cell grown with excess phosphate, (C) a cell grown with reduced phosphate, (D) a cell grown with reduced lactate, and (E) a cell grown with no phosphate. All samples were negatively stained with 1% UA. Supplementary Figure S4: CryoEM of A. fulgidus cells after three generations of reduced or elevated phosphate conditions. A) Low magnification image of a TEM grid, stained with 1% UA, of a sample grown with no phosphate. Almost no cells are present. B-E) CryoEM images of cells grown in (B) 1 mM phosphate, (C) 2 mM phosphate, (D) 4 mM phosphate (normal conditions), and (E) 9 mM phosphate. [file 4706532.f1.pdf]
